# An Overview of Methods for Reconstructing 3-D Chromosome and Genome Structures from Hi-C Data

**DOI:** 10.1186/s12575-019-0094-0

**Published:** 2019-04-24

**Authors:** Oluwatosin Oluwadare, Max Highsmith, Jianlin Cheng

**Affiliations:** 1 Department of Electrical Engineering and Computer Science, University of Missouri, Columbia, MO 65211 USA; 2 Informatics Institute, University of Missouri, Columbia, MO 65211 USA

**Keywords:** Hi-C, 3-D chromosome and genome structure, Chromosome conformation capture, 3-D genome, Contact-based modeling, Distance-based modeling, Optimization

## Abstract

Over the past decade, methods for predicting three-dimensional (3-D) chromosome and genome structures have proliferated. This has been primarily due to the development of high-throughput, next-generation chromosome conformation capture (3C) technologies, which have provided next-generation sequencing data about chromosome conformations in order to map the 3-D genome structure. The introduction of the Hi-C technique—a variant of the 3C method—has allowed researchers to extract the interaction frequency (IF) for all loci of a genome at high-throughput and at a genome-wide scale. In this review we describe, categorize, and compare the various methods developed to map chromosome and genome structures from 3C data—particularly Hi-C data. We summarize the improvements introduced by these methods, describe the approach used for method evaluation, and discuss how these advancements shape the future of genome structure construction.

## Background

After decades of research about the organization of the nucleus of the eukaryotic cell, there exists substantial evidence that the genome architecture plays a key role in nuclear functions. [1–8]. For instance, the spatial arrangement and proximity of genes has been linked to biological functions such as gene replication, regulation and transcription. [6, 9–11].

The impact of genome architecture on nuclear processes spans multiple hierarchical levels including the spatial compartmentalization of the process, the higher-order organization of chromatin and the arrangement of the genome within the nucleus. Despite the dynamic nature of their process components, processes such as transcription and DNA repair have been shown to be constrained to specific spatial locations rather than randomly dispersed throughout the nucleus. Genes tend to be more active in sparse euchromatin than dense heterochromatin, purportedly due to the impact of folding density on regulatory factor availability. The homogeneous topology of chromatin has potential to capture nuclear proteins, affecting their probability of interaction with binding sites. Small, kilo-base sized chromatin loops can localize promoters with upstream elements while larger mega-base sized loops can spatially segregate nuclear regions imposing independence on different processes.

Understanding the 3-D organization of the eukaryotic genome is essential to explain the important chromosomal activities within the cell. Hence, a fundamental question in genome and biological studies is how the spatial conformation of the chromosome in the nucleus affects a number of genetic and biological functions such as gene regulation [12, 13], gene expression [14], transcription regulation [15], DNA repair, and DNA replication [16, 17].

Early studies of chromosome conformation relied on the use of cytogenetic techniques. An example of the which is fluorescence in situ hybridization (FISH), employed to detect the presence of a specific chromosome region and the proximity between two regions in a genome sequence [18, 19]. Fluorescence in situ hybridization uses fluorescent probes that bind to specific regions of a chromosome with a high degree of sequence complementarity. Using fluorescence microscopy, the location of the loci or DNA sequence with which a probe is expected to bind may be determined. This method is especially useful, as it allows direct one-to-one estimation of genome loci proximity. However, due to technical limitations such as low-throughput, low resolution of FISH data, and probe requirements for every analysis, it is not optimal for examining multiple positions simultaneously. As a result, the method is not used when studying the organization of chromosomes at a genome-wide scale. Other microscopy techniques that have been developed to study the chromatin organization aimed at providing details about the genome positioning and activities. Some of these methods are called the super resolution microscopy strategies, as they were developed to provide imaging at a high resolution. Examples are saturated structured illumination microscopy (SSIM), stimulated emission depletion (STED), and ground state depletion (GSD**)** [20, 21]. The introduction of Stochastic super-resolution microscopy techniques such as Photo-activated localization microscopy (PALM or FPALM) and stochastic optical reconstruction microscopy (STORM) produced a different set of ways for investigating the chromatin organization [22, 23]. Generally, the microscopy techniques for studying the chromatin organization could be categorized as light and electron microscopy-based techniques. The more detailed description of the microscopy-based techniques for studying genome organization is given in the section “Genome Organization by microscopy-based techniques”.

In 2002, Dekker et al. [24] developed 3C, a high-throughput methodology that can be used to generate IFs between nearby genomic loci in a cell population. Since then, a number of 3C variants [25–27] such as 4C [28], 5C [29], Hi-C [30], TCC [31], ChIA-PET [32, 33] and, later on, single-cell Hi-C [34], have been developed to study the 3-D organization of the chromosome and genome. The development of 3C techniques has substantially benefited the study of the spatial proximity, interaction, and genome conformation of a number of cells. Today, Hi-C is the most widely used and well-known 3C variant. Using next-generation sequencing strategies such as high-throughput and parallel sequencing, Hi-C enables researchers to profile read-pair interactions on an all-versus-all basis—that is, to profile interactions for all read pairs in an entire genome. It also allows them to detect and compute the number of interactions between fragments within a chromosome—i.e., the intra-chromosome interaction frequency (IF)—or between different chromosomes—i.e., the inter-chromosome interaction frequency. Fragments, alternatively known as bins or genomic loci, are the regions to which a chromosome have been divided into. Each fragment has a defined length or size which is the number of base pair (bp) in it. The size of the fragment is determined by the resolution, e.g. a 1 MB resolution signifies that 1,000,000 bp are contained within each fragment.

The IFs obtained are commonly represented in a two-dimensional matrix, also known as a contact matrix, with rows and columns representing the number of fragments in the chromosome or genome.

The Hi-C technique is especially relevant because the IFs it yields can be used to construct 3-D chromosome and genome structures. These structures, in turn, help explain a series of events such as genome folding, gene regulations, the connection between regulatory elements and the higher-order structural features in the nucleus of a cell [1, 2, 14, 35, 36].

Within the past decade, a number of computational methods and algorithms have been proposed for the construction of chromosome and genome 3-D structures from Hi-C data. Most of these methods adopt different strategies for 3-D structure prediction, have different technical requirements for algorithms, and use different noise reduction techniques to analyze Hi-C data. In this review, we categorize these methods based on how they model IF from Hi-C data, highlight a common approach to method evaluation and validation, and finally point to the future direction and challenges of chromosome and genome 3-D structure prediction.

## Description of the Hi-C Experiment and Chromosomal Contact Map

Using next generation sequencing technology, the emergence of the Hi-C technique, an extension of 3C, has enabled the identification of the chromosome conformation at a genome wide scale [26, 27, 30, 37, 38]. Compared to other variant of the 3C technique, the Hi-C technique is the first method [30, 38] to capture chromosome conformation on a “all versus all” basis —that is, it can profile interactions for all read pairs in an entire genome. Hi-C protocol begins by using formaldehyde to crosslink the cells, which results in the covalent linking of the chromosomal loci through their protein-DNA interactions. The cross-linked chromatin segment is then cut out with a restriction enzyme, and the segment restriction ends are marked by filing in with biotin-labeled nucleotides [25, 30]. Next, the resulting blunt-end segments are ligated randomly under appropriate condition for ligation events between the cross-linked DNA segments. DNA is purified and sheared, and a biotin pull-down is performed to ensured that only the biotinylated junctions are selected for further high throughput pair-end sequencing and computational analysis. After the sequencing of the pair-reads, the generated output usually in .fastq format is mapped to a reference genome, filtered, and used to create a contact map [39]. Notable tools that support the mapping of the sequenced pair reads to generate contact map are GenomeFlow [40], Juicer [41], HiC-Pro [42], Hi-Cpipe [43], and HiCUP [44].

Interaction frequency, sometimes referred to as contact frequency, is a measure of the number of interactions between a pair of chromosomal or genomic regions in the Hi-C data [45–48]. The combined contact counts for all pairwise regions or loci may be represented as a symmetric matrix to form an IF matrix of all interacting fragments. The IF matrix is sometimes referred to as a contact matrix or contact map [30, 47]. A chromosome contact matrix is a n-by-n matrix representing the interaction of loci or chromosomal regions as captured in the Hi-C experiment [27, 30, 31, 49]. The rows and columns of the matrix correspond to the index of the equal-sized regions which partition the chromosome. The length of one equal-sized region (e.g., 1 Mb base pair) is referred to as the resolution [30]. Each entry in the matrix represents a count of read pairs that connect two corresponding chromosome regions in a Hi-C experiment [30]. Alternatively, the contacts can be represented in a 3-column sparse matrix [49], where columns 1 and 2 refer to the genomic location or the fragment number of the interacting loci and column 3 represents the IF between them.

## Polymer Model

Polymer models are based on the underlying idea that interactions between molecular subunits such as monomers result in large molecular structures known as polymers. This approach was adopted from polymer physics, a branch of statistical physics [50–52]. Polymers produced by living organisms are referred to as biopolymers. Two well-known examples of biopolymers are DNA and proteins, with nucleotides and amino acids as their monomers, respectively. Polymerization involves the combination of small molecules through chemical bonding to form a network at equilibrium called a polymer. Various authors have adopted two states of the polymer to model the architecture of chromosomal regions in a cell: the equilibrium globule [53, 54] and the fractal globule [37, 55, 56]. A characteristic feature of the equilibrium globule model is that it is highly knotted [30]. Mirny [37] has pointed out that this configuration is disadvantageous, as it restricts genomic processes such as unfolding—an important property for gene activation—or refolding [57]. Alternatively, Barbieri et al. [55] showed that polymer collapse after exposure to a topological constraint can result in the formation of a long-lived, untangled, non-equilibrium configuration state called a crumpled or fractal globule. A fractal globule is knot-free, and it is organized such that it allows for unfolding or refolding processes while in a highly compact state. Hence, the polymer exhibits a “beads-on-a-string” configuration, with beads representing monomers connected by linkers; DNA connections in eukaryotic chromatin are similarly configured. The fractal globule can be illustrated as a dense multicolor ball of yarn, where each color has its own end, but one can pull out threads with a specific color and put them back in, without disturbing the structure of the overall ball at all. This important property makes the fractal globule suitable for organizing chromatin in a cell because this topology facilitates rapid and easy unfolding, refolding [58], and large-scale opening of genome loci loop that affects and explains biological processes, e.g. the connection of distal single-nucleotide polymorphisms (SNPs) with their target genes, gene activation, gene repression, or the cell cycle [59–63].

When studying these two globules, two biophysical properties are considered: the genomic distance between two loci and the probability of contact between them. It is worth noting that genomic distance (*s*) is measured by FISH and contact probability is obtained from chromosome conformation methods such as Hi-C. The equilibrium and fractal globules yield different estimates for these properties, and therefore also varying predictions on the three-dimensional distance between pairs of loci. Lieberman-Aiden et al. [30] and Mirny [37] reports, through simulation, that equilibrium and fractal globule scaling for three dimensional-distance are *s*^1/2^ and *s*^1/3^ (s: genomic distance - number of nucleotides between two loci), respectively. Equilibrium and fractal globule scaling for contact probability are *s*^−3/2^ and *s*^−1^, respectively. As shown in [37], the properties exhibited by the fractal globule model make it more effective at fitting Hi-C data than the equilibrium globule.

**Fig. 1:**
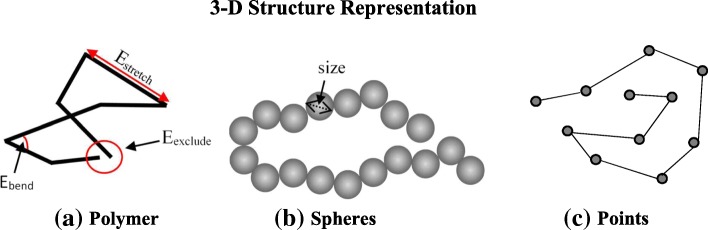
Chromosome and genome 3-D structure representation for models from Hi-C data. The different models used for representing 3-D chromosome and genome structure by various methods using Hi-C data for modeling chromosomes and genome 3-D structure. **a** polymer model, **b** spheres, **c** points

Some methods adopt the knowledge about polymer chain for chromosome structure representation by simulating a physically realistic, bead-chain polymer model of the 30-nm chromatin fiber [64, 65]. As a result, when constructing either a chromosome structure for instance, a locus for a chromosome is represented using a conventional beads-and-spring polymer model, where each bead represents a specific genomic location with well-defined initial and final genomic coordinates. Hence, viewing the chromatin fiber as a polymer model implies that conformation energies such as bending, stretching, and excluding energies of chromatin segments needs to be considered and integrated with the IF for 3-D structure reconstruction (Fig. 1a).

## Spheres and Points

An alternative structure representation model adopted by methods is representing the chromosome region or loci as series of connected spheres or interacting points. Methods using this approach presents the 3-D structure in a simplified model, where the spheres [66–68] or points [45, 46, 69, 70] are synonymous to a chromosome region or loci of a chromosome (Fig. 1b, c). Using a beads on string configuration, each bead is modeled as a spherical shape with a defined radius, and an excluded volume used to penalize overlaps between two spheres. The defined radius and the sphere volume could consequently be considered as a restraint to be satisfied during the algorithm’s 3-D structure reconstruction process. The Points representation represent the chromatin region simply as a point, with no radius nor volume, to mark the presence or absence of a loci.

## Methodologies for Chromosome and Genome 3-D Structure Reconstruction

**Fig. 2:**
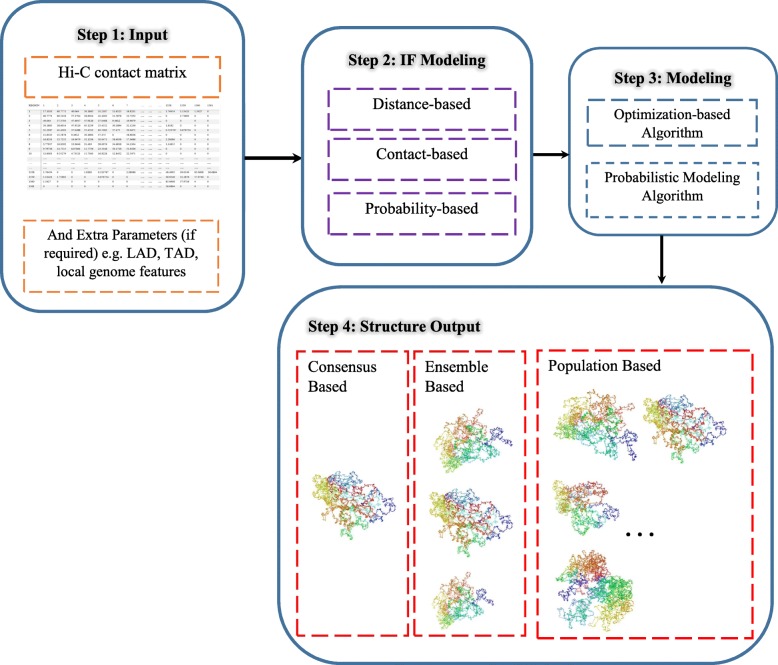
Chromosome and genome 3-D structure reconstruction workflow. A summarization of the steps for genome and chromosome 3-D structure taken by the different methods. Starting from the user input in Step 1: The input preparation, usually, Hi-C contact matrix or sometimes with extra parameters requirement. Step 2: One of the three IF modeling approach is used to represent the IF depending on the method’s algorithm. Step 3: Modeling is done using defined sampling algorithms, and Step 4, a consensus average structure or a group of structure is generated depending on the method’s structure class

The methods for chromosome and genome 3-D structure inference are categorized below based on the IF modeling adopted by them. All methods adopt a stepwise approach to achieve the 3-D structure reconstruction, and a summarization of these steps is provided in Fig. 2. In addition, the key properties of these methods are summarized in Table 1.

**Table 1:** A comparison of the methods for reconstructing 3-D chromosome and genome structure from Hi-C data

Algorithms	Year	Software Availability	Language	Structure Representation	In–built Normalization	IF Model Based	Methodology Based	Sampling Algorithm	Structure Based	Species, Coverage, and Resolution of Test 3C Data	Input
5C3D [45]	2009	No		Points	No	Distance	Optimization	Gradient Descent	Ensemble	Human*: 5C HoxA* gene cluster region.	Hi–C contact matrix
Duan et al. [66]	2010	No		Spheres	No	Distance	Optimization	IPOPT [71]– Interior–point gradient–based	Consensus	Budding yeast: Whole genome (10kb)	Hi–C contact matrix
Tanizawa et al. [67]	2010	No		Spheres	Yes	Distance	Optimization	IPOPT– Interior–point gradient–based	Consensus	Fission yeast: Whole genome (20kb)	Hi–C contact matrix
Bau et al. [72]	2011	No		Points	Yes	Distance	Optimization	IMP [73] – Monte Carlo(MC) sampling and simulated annealing with Metropolis criteria	Ensemble	Human: Chromosome 16 – 500–kb ENm008 domain (500kb)	Hi–C contact matrix
MCMC5C [48]	2011	No	Java	Points	No	Probability	Probabilistic Modeling	Markov chain Monte Carlo (MCMC) sampling using the Metropolis–Hastings algorithm	Ensemble	Human: 5C 142kb genomic region and Hi–C Chromosome 16 – 88.4 Mb region (1Mb)	Hi–C contact matrix
Meluzzi and Arya [74]	2012	No		Polymer	No	Contact	Optimization	Modified conjugate gradient algorithm and Brownian Dynamics simulation	Ensemble	Synthetic: 75kb – 270kb (3kb – 6kb)	Hi–C contact matrix
Kalhor et al. [68]	2013	No		Spheres	Yes	Contact	Optimization	Conjugate gradients and molecular dynamics with simulated annealing	Population	Human: Whole genome (1Mb)	Hi–C contact matrix
BACH [75]	2013	Yes	R	Points	Yes	Probability	Probabilistic Modeling	Gibbs sampler with hybrid MC, and adaptive rejection sampling (ARS)	Consensus	Mouse: All chromosomes (40kb)	Hi–C contact matrix and local genomic features (restriction enzyme cutting frequencies, GC content and sequence uniqueness) as input
ChromSDE [76]	2013		Matlab	Points	No	Distance	Optimization	Linear and Quadratic Semi–definite programming(SDP)	Consensus	Mouse and Human: Chromosome 13 (200kb – 1Mb 40kb(chr13:21Mb–25Mb))	Hi–C contact matrix
AutoChrom3D [77]	2013	Yes	Perl	Points	Yes	Distance	Optimization	Non–linear constrained optimization	Consensus	Human: 500kb – 1MB (8kb)	Hi–C contact matrix
PASTIS [78]	2014	Yes	Python	Points	No	Distance and Probability	Optimization(MDS1, MDS2) and Probabilistic Modeling (PM1,PM2)	IPOPT – interior point filter algorithm	Consensus	Mouse: All chromosomes (100kb – 1Mb, 20kb –chr1-19)	Hi–C contact matrix
ShRec3D [79]	2014	Yes	Matlab	Points	No	Distance	Optimization	Shortest-path Floyd-Warshall algorithm	Consensus	Human: Chromosome 1 – 30Mbp region (3kb - 150kb)	Hi–C contact matrix
MOGEN [49, 80]	2014	Yes	Java	Points	No	Contact	Optimization	Gradient descent	Ensemble	Human: All chromosomes and whole genome (200kb - 1Mb)	Hi–C contact matrix
FisHiCal [81]	2014	Yes	R	Points	Yes	Distance	Optimization	SMACOF algorithm [82]	Consensus	Human: Whole genome (1Mb)	Hi–C contact matrix
InfMod3DGen [64]	2015	Yes	Matlab	Polymer	No	Distance	Probabilistic Modeling	Gradient ascent	Ensemble	Yeast: All chromosomes –12.1Mb genome (10kb)	Hi–C contact matrix
Gen3D [83]	2015	Yes	C++	Points	No	Contact	Optimization	Adaptation, Simulated annealing and Genetic algorithm	Consensus	Human: All chromosomes (1Mb)	Hi–C contact matrix
MBO [84]	2015	Yes	Matlab	Points	No	Distance	Optimization	Manopt – manifold optimization	Consensus	Mouse: Chromosome X (50kb - 600kb)	Hi–C contact matrix
TADbit [85]	2016	Yes	Python	Spheres	Yes	Distance	Optimization	Simulated Annealing and Monte Carlo Sampling	Ensemble	Drosophila Fly: 52Mb region (10kb)	Hi–C contact matrix
HSA [47]	2016	Yes	R	Points	Yes	Distance	Optimization	GLM framework with Hamiltonian dynamics with simulated annealing	Consensus	Human and Mouse: All chromosomes (25kb - 1Mb)	One or more raw contact maps or normalized Hi–C contact matrix.
Chromosome3D [46]	2016	Yes	Perl	Points	No	Distance	Optimization	Distance Geometry Simulated Annealing	Ensemble	Human: All chromosomes (500kb - 1Mb)	Hi–C contact matrix
PGS [65, 86]	2016	Yes	Python	Spheres	Yes	Probability	Probabilistic Modeling	Simulated annealing/molecular dynamics	Population	Human: Whole genome (50kb - 1Mb)	Raw Hi–C contact matrix and a TAD file in bed format
tRex [87]	2016	Yes	R	Points	Yes	Probability	Probabilistic Modeling	MCMC sampling using the Metropolis–Hastings algorithm/Gibbs sampler, Hamiltonian MCMC	Ensemble	Human: Chromosome 14 and 22 (1Mb)	Hi–C contact matrix and a vector of covariates (e.g. fragment length, GC content, and mappability score)
3D–GNOME [88, 89]	2016	Web server	C++, Javascript, PHP, Python, R	Polymer	Yes	Distance	Optimization	Monte Carlo-based simulated annealing	Consensus	Human: All chromosomes (Multiscale 1-2Mb, PET (1–10kb))	A seven or eight columns bedpe (paired–end BED format) file containing the locations and strengths of long range contact points. Use of ChIA-PET data is recommended
LorDG [69]	2016	Yes	Java	Points	No	Distance	Optimization	Gradient ascent	Ensemble	Human: All chromosomes and whole genome (500kb –1Mb)	Hi–C contact matrix
ISDHiC [90]	2016	No	C, C++, Python	Spheres	No	Distance	Probabilistic Modeling	MCMC sampling using Hamiltonian MC	Ensemble	Mouse: Chromosome X (50kb, 500kb)	Hi–C contact matrix
Chrom3D [91]	2017	Yes	Perl	Spheres	No	Contact	Optimization	Monte Carlo-Optimization using the Metropolis–Hastings algorithm with simulated annealing	Ensemble	Human: Whole genome (TAD)	Hi–C contact matrix and LAD information
miniMDS [92]	2017	Yes	Python	Points	No	Distance	Optimization	MDS approximation algorithms and Kabsch algorithm	Consensus	Human: Whole genome (10kp-100kb)	Hi–C contact matrix
3DMax [70]	2018	Yes	Java, Matlab	Points	No	Distance	Optimization	Gradient ascent	Ensemble	Human: All chromosomes (1Mb)	Hi–C contact matrix
GEM [93]	2018	Yes	Matlab	Polymer	No	Contact	Optimization	Adaptive gradient descent method	Ensemble	Human: Chromosome 13 and 14(1Mb), Chromosome 1 (250 kb: 130Mb-180Mb region ), Yeast : Chromosome 6 (10kb),	Hi–C contact matrix
GEM–FISH [94]	2018	Yes	Matlab	Polymer	No	Contact	Optimization	Gradient descent	Consensus	Human: Chromosomes 20, 21, 22, and X (TAD)	Hi–C contact matrix and FISH data
SIMBA3D [95]	2018	Yes	Python	Points	No	Probability	Probabilistic Modeling	BFGS mehtod with analytical gradient	Ensemble	Mouse: All chromosomes (100kb)	Hi–C contact matrix
ShRec3D+ [96]	2018	No		Points	No	Distance	Optimization	Floyd-Warshall algorithm	Consensus	Human and Mouse: All chromosomes (1Mb)	Hi–C contact matrix
EVR [97]	2018	Yes	C, Python	Points	No	Distance	Optimization	Error-Vector Resultant algorithm	Consensus	Bacteria: All chromosomes (10kb)	Hi–C contact matrix
Hierarchical3DGenome [98]	2019	Yes	Java	Points	No	Distance	Optimization	Gradient ascent and hierarchical modeling	Ensemble	Human: All chromosomes (1kb - 5kb)	Hi–C contact matrix and File containing identified TADs

Each column denotes the key properties of each method. Algorithms column denotes the 3-D structure reconstruction method’s name or acronym, Year column denotes the publication year, Software Availability column denotes the availability of an open-source software for a method, Language column denotes the programming language that the software was implemented in, Structure Representation column denotes the structural representation used by a method— which could either be as polymer, spheres, or points, detailed explanation for each is provided above, In–Built Normalization column denotes if a normalization step is in-built into a method’s algorithm, Interaction Frequency Model Based column denotes a method’s class based on its input IF modelling, Methodology Based column denotes a method’s class based on its chromosome and genome 3-D reconstruction methodology, Sampling Algorithm column denotes the sampling or one of the sampling algorithms used by a method, Structure Based column denotes a method’s class based on the structure generated—consensus-based methods generate a single representative structure for the entire Hi-C dataset or for single-cell Hi-C data, ensemble-based methods generates a variety of 3-D structures while using the same input H-C data to adequately simulate the heterogeneity of the Hi-C data, population-based methods generates a population of individual 3-D structures of genome that is as a whole statistically consistent with true configuration of the input Hi-C data, Species,Coverage, and Resolution of Test 3C data column denotes the size, resolution, species of Hi-C data used in a method’s manuscript, and Input column denotes a method’s input data format. Even though these methods may be tested only on the Hi-C data of some species, most, if not all, the methods should be applicable to any Hi-C contact data of any species formatted according to the input requirement

### Distance-Based Methods

Over the years, a number of approaches have been proposed for chromosome 3-D structure inference from Hi-C contact data. A group of these methods involve a two-step process: (1) IF is converted to distance, ultimately defining the problem of 3-D genome or chromosome structure reconstruction as a problem of converting distances into 3-D coordinates; and (2) non-linear optimization is subsequently applied to the problem in order to find the genomic coordinates that satisfy converted distances. The most notable differences between these proposed methods are: (1) the way in which IF is converted into distance, and (2) the optimization technique used to infer the 3-D structure from loci distance. The aim of a distance-based modeling is to create a map that shows the relative spatial positioning of a number of objects whose inter-point distance is known. Additionally, representing chromosome structure prediction as a distance-based modeling problem is tempting because methods based on distances are simple and clear: there is no ambiguity regarding metric definition and proximity between objects can eventually be derived. In relation to 3-D genome structure prediction, the distance-based approach makes it easier to handle a large spectrum of modeling problems at different Hi-C data resolutions.

The distance-based approach attempts to reproduce the original metric or distance as accurately as possible. The earliest application of the metric multi-dimensional scaling (MDS) [82, 99] to chromosome 3-D conformation construction, known as 5C3D [45], assumed that the relationship of IF to distance between DNA fragments or loci follows an inverse relation; it then used an optimization approach to find the best 3-D conformation through a misfit objective function of the converted distance and the 3-D Euclidean distance between points. While this method was applied to the 5C variant of 3C data, it could be applied to Hi-C datasets as well. Similarly, in their work based on yeast 3-D genome structure reconstruction, Duan et al. [66] designed a metric that estimated the corresponding Euclidean distance from the mean of the curves obtained from two restriction enzyme libraries for each contact frequency. To aid modeling and ensure that intra- and inter-chromosomal features (e.g., centromeres), distance, and properties were satisfied [66, 67], researchers introduced a series of constraints such as minimum and maximum distances between adjacent beads, minimum distances between pair beads to avoid overlapping and clashes, specific positioning of RNA coding regions, telomeres, and centromeres to guide the construction of the 3-D model; this constituted an improvement over the previous method. Duan et al. used IPOPT [71], an open-source software for nonlinear constrained optimization problems, to minimize the objective function; this ensured that the predicted coordinates of two interacting loci, from which the distance between said loci in the 3-D structure is derived, closely matched the expected distance obtained from IF. Tanizawa et al. [67] developed a method similar to [66] to construct the 3-D structure of the fission yeast genome.

Although Lieberman-Aiden et al. [30] showed that IF can be used to determine the spatial distance between interacting loci, certain factors regarding this conversion are still worth considering. As shown by [76, 100–102] in their work, the IF-distance correlation might vary from one dataset resolution to another, and from one organism to another. Hence, an efficient method is required for a distance-based approach to generate a more reliable distance estimate from IF data. To solve this problem, Zhang et al. [76] made two novel propositions for the two-step genome structure prediction pipeline. First, they used a modified version of the golden section search method [103] to determine the best scale parameter, conversion factor (α), to convert IF to its approximate distance equivalent: *D*_*ij*_ ∝ *F*_*ij*_^−*α*^; this ensures that an appropriate conversion factor is obtained for each dataset. Secondly, for the 3-D structure prediction from a distance matrix, they presented an algorithm called ChromSDE (Chromosome Semi-Definite Embedding). Unlike earlier methods, ChromSDE relaxed the optimization problem to a semi-definite programming (SDP) problem. The proposed approach to IF-distance conversion defined by Zhang et al. introduced a new convention for defining the IF-distance relationship, followed by a series of distance-based algorithms that were subsequently developed.

According to Yaffe and Tanay [104], raw Hi-C data obtained from 3C experiments may contain numerous systematic biases, such as GC content, length of restriction fragments, and mappability between fragments. *L*ong-range frequencies are typically noisy and unreliable; this represents a substantial drawback for the construction of 3-D chromosome and genome structures*.* In order to overcome these limitations, a number of methods have been developed to pre-process Hi-C data through normalization [9, 42, 104–108] before using the data for 3-D reconstruction. Alternatively, certain algorithms for 3-D structure construction incorporate bias removal. Peng et al. [77] proposed a normalization approach to reduce experimental sequencing depth bias, which affects the IF yielded by Hi-C data and makes it hard to compare structures from data obtained from different experiments. The method, called AutoChrom3D, provides an automated pipeline for 3-D modeling, enabling structural comparison at various data resolutions. Two linear transformations were used to determine the frequency-distance correlation, and structure was predicted through nonlinear constrained optimization. Shavit et al. [81] designed an MDS-based optimization approach that used FISH distance to guide the conversion of IF to Hi-C loci distances; this approach aimed to reduce noise, improve the data quality, ensure the consistency of data used for 3-D structure construction, and cover key functionality features in the Hi-C and FISH datasets, which will eventually overlap if these features are vital. Zou et al. [47] designed a flexible algorithm capable of handling biases introduced by restriction enzymes during Hi-C data sequencing. Restriction enzymes are known to have various cutting sites across the genome, so combining different Hi-C tracks provides further information about genomic loci for modeling. The tool developed by Zou et al., called HSA, takes advantage of the uniqueness of the contact map obtained from different restriction enzymes in Hi-C experiments; it creates a generalized linear model through an iterative algorithm that combines simulated annealing and Hamiltonian dynamics. By using HSA, Zou et al. discovered that the obtained 3-D structure fits the contact map obtained from different restriction enzymes. Bau et al. [72] performed a log transformation and the Z-score computation to normalize the contact counts. They converted observed interactions between loci to points and spatial restraints, and used the Integrative Modeling Platform (IMP) [73] to produce possible confirmations that satisfies their defined constraints and maximizes their structure to fit the IF data. Each loci was first represented as a point connected by a “string” to create a pairwise interaction in which the length of the string depended on the number of interactions between the loci.

To date, a number of other distance-based methods have been developed. These algorithms create 3-D models by first converting contact frequency to distance [9, 46, 69, 70, 77, 88, 97, 109, 110] and then apply optimization to predict chromosome structure. Usually, these methods perform chromosome 3-D reconstruction by first defining a random 3-D structure; this structure coordinates are then updated by an objective function that is iteratively optimized until a convergence condition is satisfied. Chromosome3D [46], applied a modified version of the distance geometry simulated annealing (DGSA) based method for chromosome and genome 3-D structure reconstruction from Hi-C data. The DGSA method has been popularly used for protein structure construction over the years and implemented in the Crystallography & NMR System (CNS) suite [111, 112]. The Hi-C distances are used as restraints for the defined simulated annealing (SA) optimization pipeline. SA is carried out through multiple steps of temperature change until the defined structure energy is optimally minimized. Because Chromosome3D uses one of the rigorously tested approaches in protein structure to inferring chromosome and genome 3-D structure, it is reliable and robust against noise in Hi-C data.

LorDG [69] introduced a novel method to address inconsistent chromosomal contacts generated from multi-cell Hi-C data. It used a nonlinear Lorentzian function as the objective function—to enforce the satisfaction of consistent restraints, which is resistant against noisy distance restraints. Unlike the square error function that is susceptible to outliers, LorDG aims to maximize the satisfaction of realistically satisfied restraints rather than unsatisfiable noisy ones. The objective function is optimized by the highly scalable adaptive step-size gradient descent method. Its resilience against noisy contacts and scalability make it a suitable method for constructing the structure of the entire genome involving noisy inter-chromosomal contacts. 3DMax [70] defined a maximum likelihood objective function for chromosome 3-D structure inference from Hi-C data. It is based on the simplified assumption that the contact data is normally distributed and that each Hi-C data point is conditionally independent given a structure. A log likelihood objective function for chromosome structure reconstruction was defined in order to determine the structure that maximizes the likelihood function. 3DMax uses a variant of gradient ascent called Adagrad [113] that adapts the learning rate to each objective function parameter automatically to regulate its learning rate. 3DMax is robust against noise and structural variability, and it is computationally fast and memory efficient.

miniMDS [92] and Hierarchical3DGenome [98] are the distance-based algorithms that reconstruct high-resolution 3-D models at the topologically associating domain (TAD) level. Eventually, these TAD models are assembled to form a complete, high-resolution 3-D chromosomal structure. After the assembly of TAD models, Hierarchical3DGenome uses the contacts between all regions in a chromosome to further refine the assembled whole chromosome model, which leads to high-resolution (e.g. 5 KB) models of good quality.

The conformational space of a chromosomal structure is large, given that Hi-C data are drawn from a population of cells, each with its own independent and unique 3-D structure. Hence, an ensemble of predicted structures obtained through so-called ensemble-based modeling appears to provide a better representation of chromosomal structure than a single structure obtained through consensus modeling. Unfortunately, like Hi-C data at large, this dataset contains a number of biases: the fact that it is noisy, coupled with other technical factors, makes it extremely difficult to determine the various unique 3-D structures of cells used in Hi-C experiments. Due to the drawbacks involved in using multi-cell Hi-C data, studying single-cell Hi-C data has become increasingly relevant [34]. In particular, it does not require designing an algorithm to satisfy the variability of each cell used in the Hi-C experiment. As expected, single-cell Hi-C datasets are sparser than multi-cell Hi-C datasets. Hence, conventional distance- and restraint-based methods are not suitable for 3-D structure reconstruction based on these data. Carstens et al. [90] extended Rieping et al.’s [114] Bayesian probabilistic framework to statistically infer ensembles of 3-D chromosome structures from single-cell Hi-C data using MCMC sampling. They combined single-cell Hi-C contact information with FISH data and a coarse grained model of the chromatin fiber. Lesne et al. [79] formulated a two-step algorithm known as “shortest-path reconstruction in 3-D” (ShRec3D), which combines the shortest-path distance between two points from graph theoretic methods with MDS to achieve chromosome reconstruction*.* This method is designed for both multi-cell and single-cell Hi-C data. In the case of single-cell Hi-C data, instead of distances between two points, binary numbers signify the presence or absence of interaction. ShRec3D+ [96] extended Lesne et al.’s algorithm by using a golden-section algorithm (an approach similar to Zhang et al. [76]) with an adaptable distance conversion factor for different Hi-C chromosome datasets. Wang et al. [64] proposed a method that combined knowledge of the conformational energy model of a chromatin structure and a Bayesian inference approach. They represented the chromosome structure as a polymer model with a conformational energy, and integrated the IF data as input for an expectation maximization based algorithm under a Bayesian like framework. They took advantage of the prior information about the conformation energy to construct a Bayesian inference of the chromatin structure. An approach proposed by Paulsen et al. [84] employed manifold-based optimization (MBO), which is basically the application of optimization techniques to the manifold of positive semi-definite matrices of fixed rank [115]. Paulsen et al. reported that MBO is capable of generating a consensus 3-D chromosome structure consistent with the original contact map.

Another approach for solving the distance-based problem is called non-metric multidimensional scaling (NMMDS), which assumes that only distance ranks are known; distances themselves are not provided. The method aims to yield a map of these ranks [116, 117]. Using this approach, Ben-Elazar et al. [118] developed a method for structure prediction based on the hypothesis that a pair locus A with a higher IF is closer in 3-D space than any other locus pair B with a lower frequency. Varoquaux et al. [78] also proposed an optimization method to solve the NMDS problem by minimizing the Shepard-Kruskal scaling cost function [119].

### Contact Based Methods

Certain methods do not convert IF but use it directly for modeling. These methods are regarded as contact-based methods [15, 80, 83, 91, 93]. MOGEN [49, 80] used contact directly and designed an optimization-based approach that relied mostly on Hi-C intra- and inter-chromosomal contact data to build an ensemble of 3-D conformations for genome and chromosome structures. The contact-based optimization is carried out by the adaptive step-size gradient descent/ascent method that is highly scalable and therefore is well suited for large-scale genome structure modeling. MOGEN does not require two contacted regions to satisfy a specific distance as the distance-based approach does. Instead it only tries to make the distance between the two contact regions below a threshold (i.e. in contact). MOGEN is capable of producing ensemble models that are highly consistent with each other. MOGEN is also robust against noise in the data, particularly the noise in inter-chromosomal contacts, and therefore it is able to build 3-D structures of large genomes such as the entire human genome. Gen3D [83] used a series of meta-heuristic algorithms (e.g. genetic algorithms and simulated annealing) to infer 3-D structure from IF. Zhu et al. [93] proposed a manifold-based framework called GEM, which first uses IF to create an interaction network representing the spatial organization of the loci from Hi-C data. Zhu et al.’s aim was to use a manifold learning algorithm to uncover the low-dimensional (3-D) geometry embedded in a high-dimensional (Hi-C) space, while satisfying certain defined conformation energy requirements. An improvement over this method integrates Hi-C data with FISH data for 3-D structure inference [94]. To ensure the modeling of realistic structures consistent with cellular organization, Paulsen et al. [91] introduced Chrom3D, a genome-modeling algorithm that combines Hi-C and Lamina-associated domain (LAD) information from ChIP-seq data to generate an ensemble of 3-D genome structures in which loci and TAD positioning and interaction requirements are satisfied.

On the other hand, certain methods convert contact frequency into defined spatial restraints. As is the case with distance-based approaches, these restraints are satisfied through an optimization method. In their seminal study, Kalhor et al. [68] developed a 3C variant known as tethered conformation capture (TCC), aimed at increasing the signal-to-noise ratios in conformation capture experiments. This is relevant because it allows for a more accurate representation of IF, especially for genome structure analysis, where low inter-chromosomal interactions are recorded using existing approaches. Using TCC data, researchers proposed a novel modeling approach whereby a variety of genome structures were generated. This approach, called population-based modeling, produces a population of structures representative of genomic configuration and consistent with contact probability. Serra et al. [85] followed certain constraints in order to transform IF into spatial restraints; for instance, consecutive and non-consecutive loci were treated differently. As in the case of Bau et al. [72], these restraints were satisfied by using the IMP.

### Probability Based Methods

Methods in this category define a probabilistic measure for contact frequency, hence their name. Using a probabilistic approach to model 3-D structures has a number of advantages; key among them is that such an approach allows uncertainties in experimental Hi-C data to be easily considered through probabilistic representation. In addition, statistical calculations of specific structural properties or noise sources can be carried out. Due to the fact that Hi-C data are drawn from cell populations, IF can be considered as an average; most probability-based methods assume that an ensemble of structures underlies a contact map. In addition, they consider the problem of 3-D structure inference as either a Bayesian inference problem or a maximum likelihood problem. However, some probabilistic modeling may be more time consuming than other methods.

Rousseau et al. [48] developed the first method in this category, called MCMC5C. They defined a probabilistic model of IF and used a Markov chain Monte Carlo (MCMC) sampling to generate an ensemble of structures. MCMC5C through a Gaussian model based on Hi-C data, whose variance was estimated using an improvised approach. A MCMC sampling-based algorithm was selected over alternatives methods because of its inherent ability to estimate the distribution of various structural properties. As previously mentioned, raw Hi-C data contain a number of systematic biases such as GC content, restriction enzyme cutting frequency, and sequence uniqueness [104]. These factors all need to be considered when designing a 3-D genome reconstruction method. To overcome these limitations, Hu et al. [75] proposed two Bayesian models for 3-D genome structure reconstruction from Hi-C data. Their methods combined bias removal with 3-D genome structure construction. They corrected known biases and used a Poisson model to fit contact data, an improvement over MCMC5C when it came to estimating the Gaussian variance. Varoquaux et al. [78] also defined a probabilistic model of IF. Similar to the model defined by Hu et al., it defined the structure inference problem as a maximum likelihood problem and used an optimization method to solve it.

A typical drawback of high-resolution Hi-C data is the sparsity of long-range contacts on the contact matrix and the high proportion of zero-contact counts between loci in the matrix. Hence, certain existing methods might be incapable of modeling at a higher resolution. Park and Lin [87] proposed an algorithm that is robust to resolution specification and corrects known systematic biases. They modeled the contact count using a Poisson distribution and addressed excess zero problems in high resolution datasets. They suggested that these problems could be solved by adjusting the Poisson distribution adopted for modeling.

Nagano et al. and Stevens et al. [34, 120, 121] applied a simulated annealing technique to sample single-cell datasets, while sometimes using contacts as distance restraints at different data resolutions. A novel study by Tjong et al. [86] has proposed a population-based modelling approach called PGS. Different from the ensemble-based approach—where a variety of structures with different variabilities are generated to simulate the heterogeneity of cells in the Hi-C experiment—the population of genome structures generated by PGS is consistent with the normalized contact probability matrix. Tjong et al. have formulated a probabilistic framework that uses an EM algorithm with constraint assignment at the E step and optimization of the structure population through simulated annealing and conjugate gradient descent at the M step. This method takes advantage of other external experimental data, such as lamina information for improved modeling. Rosenthal et al. [95] proposed an approach to recover missing contacts in single-cell Hi-C contact maps by filling missing parts with structures obtained from the corresponding cell populations, while imposing certain penalties on the generated structures.

## Correcting Biases in Hi-C Data by Data Normalization

As is the case for most sequencing experiments, raw Hi-C data contain several systematic biases that could potentially affect the 3-D genome reconstruction. An inexhaustive list of these systematic biases include GC content, distance between restriction sites, restriction enzyme cutting frequency, sequence uniqueness, and experimental artifacts [104]. In a Hi-C experiment protocol, a minimum of 25 million cells was used to produce a Hi-C library [27, 30, 38, 69] with the goal of analyzing the contact frequencies between genomic sites in a cell population. One of the reasons for using a population of cells in Hi-C experiments is more sequence reads can be produced from a population of cells than a single cell.

The number of paired-end reads linking two genomic regions is interpreted as the interaction frequency between two genomic regions. This implies that a higher interaction frequency on a contact map means that a higher read count was observed, and that the two regions are spatially close to each other. However, many of these systematic biases affect the observed Hi-C read counts for two interacting regions (or fragments) on a contact matrix [106]. Hence, when these biases are left unhandled, the 3-D model construction is predicated on inaccurate information and consequently may be adversely affected. Additionally, if the effect of duplication, deletion, inversion and ploidy is significant in the pair reads, this could cause a direct effect on the number of paired-end reads linking two genomic regions which will alter the derived contact map. Because the Hi-C contact data is used for 3-D genome modeling, the level of correctness of the Hi-C data largely determines the accuracy of the generated model.

To overcome these limitations, most 3-D reconstruction methods apply normalization methods that focus on removing biases introduced by experimental procedures and by intrinsic properties of the genome to preprocess the data [9, 42, 104–108]. With the application of a normalization and pre-processing technique before 3-D genome reconstruction, the noise and systematic biases introduced by external factors, such as DNA shearing, and cutting, during the Hi-C experiment makes the Hi-C data more suitable for chromosome/genome 3-D structure reconstruction. Alternatively, some probability-based reconstruction methods handle the noise and biases differently by taking the biases into consideration in their algorithm design [75].

A common problem observed in some Hi-C data is the omission of the contact frequency of some genomic positions in the contact matrix. When this occurs, the reconstructed 3-D model from this data varies across the different tools due to difference in the way the methods represent omissions in their 3-D model. Generally, this leaves some doubt about which 3-D model is better when this occurs.

## Validation and Evaluation

According to the literature on chromosome and genome 3-D construction methods, algorithms are most often validated by a simulated dataset to assess their reconstruction ability, the consistency with the Hi-C data, known genome and chromosome structural features [49], or Fluorescence in situ hybridization (FISH) data. In the simulation case, most methods use a 3-D polymer model meant to serve as a gold standard model with which to compare the final 3-D reconstructed structure. A set of chromosomal contact data is then simulated from this structure, and a certain degree of Gaussian noise is often added to the data as well. The noise is usually added to assess the methods’ responsiveness and accuracy to noisy data. Eventually, the algorithms’ ability to reconstruct the true model is tested. A commonly used synthetic dataset is the one generated by Trussart et al. [122]. Trussart et al. created a series of simulated Hi-C contact matrices in which genomic architectures are pre-defined, and the noise level and structural variability (SV) are both simulated.

FISH provides a powerful tool for identifying the location of a DNA sequence. It is used to study the 3-D organization of chromosomes and genomes and determine the proximity of a gene relative to other genes through the use of fluorescent probes [123]. It has been determined to be much more accurate, simple, and reliable than all other molecular profiling techniques [124]. Hence, it is often used to determine the distance between loci in a genome and for single-cell analysis of gene and loci positioning [125–128]. However, its major limitations are low throughput and resolution at higher scales, such as the entire genome or an ensemble of cells. Nonetheless, FISH data can be used to validate the distance between loci in a reconstructed 3-D structure at a lower scale. Given that the FISH method is considered reliable, it is useful in the study of chromosomal and genomic 3-D spatial organization when loci in the structure being evaluated are physically proximal.

Once the structure construction is complete, a method is often needed to assess its accuracy. The most common approach to structure evaluation is to calculate the Pearson correlation coefficient (PCC), the Spearman correlation coefficient (SCC), or the root mean square error (RMSE) of the distance representation of the Hi-C data and the Euclidean distance of the 3-D chromosomal structure. Since these metrics are obtained for distance, they are sometimes referred to as the distance Pearson correlation coefficient (dPCC), the distance Spearman Correlation Coefficient (dSCC), and the distance root mean square error (dRMSE). The value of dSCC and dPCC is in the range of − 1 to + 1, with higher values being preferable. In the case of dRMSE, on the other hand, a lower value is preferred. The latter may vary between 0—which signifies no difference between distances—and a large upper limit dependent on the number of fragments in the structure being compared when they are completely different. The dRMSE is also an appropriate metric to assess the similarity between 3-D structures. In order to do so, a linear transformation that includes translation, orthogonal rotation, and rescaling is performed on one of the structures, so that they are at the same 3-D-coordinate scale as in [49].

Let the pairwise distance between Hi-C data IF be represented by the vector {D_i_, …, D_n_} and the Euclidean distance between loci in a 3-D chromosome model be represented as {ED_i_, …, ED_n_}, where n is the number of loci pairwise distances. The dSCC, dPCC, and dRMSE can be computed as shown below:

1. The dPCC is defined as:
  $$ \mathrm{dPCC}=\frac{\sum_{i=1}^n\left({D}_i-\overset{\acute{\mkern6mu}}{D}\right)\left({ED}_i-\overset{\acute{\mkern6mu}}{ED}\right)}{\sqrt{\sum_{i=1}^n{\left({D}_i-\overset{\acute{\mkern6mu}}{D}\right)}^2{\sum}_{i=1}^n{\left({ED}_i-\overset{\acute{\mkern6mu}}{ED}\right)}^2}} $$

where:

- *D*_*i*_ and *ED*_*i*_ are single distance samples indexed with i,
- *n* is the number of loci pairwise distances,
- $\overset{\acute{\mkern6mu}}{D}$ and $\overset{\acute{\mkern6mu}}{ED}$ represent sample means. $\overset{\acute{\mkern6mu}}{D}=\frac{1}{n}{\sum}_{i=1}^n{D}_i$, $\overset{\acute{\mkern6mu}}{ED}$ = $\frac{1}{n}{\sum}_{i=1}^n{ED}_i$ .

(2) The dSCC is defined as:

$$ \mathrm{dSCC}=\frac{\sum_{i=1}^n\left({A}_i-\overset{\acute{\mkern6mu}}{A}\right)\left({B}_i-\overset{\acute{\mkern6mu}}{B}\right)}{\sqrt{\sum_{i=1}^n{\left({A}_i-\overset{\acute{\mkern6mu}}{A}\right)}^2{\sum}_{i=1}^n{\left({B}_i-\overset{\acute{\mkern6mu}}{B}\right)}^2}} $$

dSCC is calculated by converting distance variable *D*_*i*_ and *ED*_*i*_ into ranked variables *A*_*i*_ and *B*_*i i*_, and then, computing the dPCC between the ranked variables. Hence, the pairwise distances *D*_*i*_ and *ED*_*i*_ are converted into ranked variables *A*_*i*_ and *B*_*i*_ respectively,

where:

- *A*_*i*_ and *B*_*i*_ are the ranks of two distances, *D*_*i*_ and *ED*_*i*_ respectively.
- $\overset{\acute{\mkern6mu}}{A}$ and $\overset{\acute{\mkern6mu}}{B}$ represent sample means of rank. $\overset{\acute{\mkern6mu}}{A}=\frac{1}{n}{\sum}_{i=1}^n{A}_i$, $\overset{\acute{\mkern6mu}}{B}$ = $\frac{1}{n}{\sum}_{i=1}^n{B}_i$ .

(3) The dRMSE is defined as:

$$ \mathrm{dRMSE}=\sqrt{\frac{1}{n}\sum {\left({D}_{ij}-{ED}_{ij}\right)}^2} $$

- where *D*_*ij*_ and *ED*_*ij*_ represent the pairwise distance between loci i and j of the Hi-C IF data and 3-D structure Euclidean distance
- *n* is the number of loci pairwise distances.

## Microscopy-Based Techniques for Studying Genome Organization

Although this review highlights the methods for genome structure reconstruction from Hi-C data, it is noteworthy to examine the complementary imaging methods used for studying the genome organization before and after the emergence of high-throughput sequencing techniques. For many years, the structure of the genome has been studied through various microscopy techniques [23, 129–134] which can be broadly divided into electron and light microscopy.

The light microscope alternatively referred to as the optical microscope is a well-known research tool that uses visible light to detect small objects. Over the years, light microscopy has greatly enhanced the study of the events and the structural details in the cell nucleus. However, the light microscopy techniques have a well-known limitation for being unable to overcome the diffraction barrier. As a solution to this, several strategies have been proposed to bypass the diffraction barrier of light microscopy and increase resolution. These strategies are called the super resolution microscopy strategies. They include saturated structured illumination microscopy (SSIM), stimulated emission depletion (STED), and ground state depletion (GSD) [20, 21]. The introduction of Stochastic super-resolution microscopy techniques such as Photo-activated localization microscopy (PALM or FPALM) and stochastic optical reconstruction microscopy (STORM) ushered in a new wave of discovery about the genome organization [22, 132, 135]. These techniques allow obtaining images at a higher resolution because they are not limited by the diffraction barrier in optical microscopy. These methods use florescent probes for imaging in multiple colors and support the selection of many fluorescent molecules at a very high resolution to build point by point images that display the relationship between points [135]. The STORM and PALM techniques elevated the visualization of the genome structure to an incredibly high resolution. Ricci et al. [136] used the STORM technique to visualize the chromatin fiber structure of different cells at a nanoscale resolution, single cell level, which revealed nucleosome groups along the chromatin fiber which they called “nucleosome clutches”

One type of light microscopy technique, fluorescence microscope, uses fluorescence and phosphorescence to study the properties of and visualize an object or cellular component of a cell. The fluorescence microscopy technique uses a light intensity that is significantly higher than other light microscopy techniques [137–139]. Fluorescence microscopy technique is effective at visualizing fluorescent dyers stains [140–142] as well as autofluorescence cellular structures i.e. biological structures which naturally emit fluorescent light [139]. The technique is also used when studying the expression and the localization of proteins using fluorescent antibodies in a biochemical strategy called immunofluorescence. The fluorescence dyers stains are used to determine cellular structure and identify specific targets of interest within a cell. A major limitation of the fluorescence microscopy technique is photobleaching. Photobleaching causes the fading of the dye or a fluorophore molecule making it lose its fluoresce properties, hence rendering the protein molecules or object invisible. Fluorescence recovery after photobleaching (FRAP) [143, 144], and Fluorescence loss in photobleaching (FLIP) [145] analysis are fluorescence microscopy technique used to examine diffusion and molecular movement respectively in a cell. FLAP, FRET and FLIM are also advanced fluorescence microscopy techniques that are used in biological and biomedical research [146].

For some time, the 3-D genome organization was largely discovered through the fluorescent in situ hybridization (FISH) technique. The FISH [2, 18, 19] technique uses a florescence probe to detect specific DNA (or RNA) sequences or selected genome loci in single cell nuclei by light microscopy. Today, there are different types of FISH, each with their specialized function e.g. DNA-FISH, RNA-FISH, cryo-FISH e.t.c. [147]. These variants are more prolific than FISH because of their accuracy, and reliability. FISH techniques allow the conceptualization of the arrangements of genetic materials in the cell nucleus. The FISH technique has revealed that the chromosomes occupy discrete territories in the cell nucleus, referred to as chromosome territories (CT) [2, 148], CT intermingle significantly in the nucleus of human cells [149], the influence of gene density and transcription on chromosome organization in the nucleus [150, 151], and the genome organization in the nucleus based on the partitioning of the chromosomes regions according to the gene distribution [152, 153]. The findings have increased the understanding about the genome architecture and behavior in the nucleus of the cell. However, the FISH technique can only be used to examine predetermined regions in cells. To resolve this, a fully automated FISH-based imaging pipeline called High-throughput imaging position mapping (HIPMap) was developed to perform high-precision, high-throughput, automated fluorescent in situ hybridization imaging of the spatial location of genome regions at large scale [154].

Electron microscopes uses a beam of high energy electrons to examine objects and obtain information about that object or a specimen. This provides the information about the surface characteristics, composition of the elements that makes up the object, the particles within the objects, and the arrangement of the atoms within the object. It was developed due to inability of the light microscope to examine the information about structure of smaller objects. The development of the electron microscope improved the resolution so that tiny objects e.g. atom can be observed under this microscope. To examine objects only observable at the higher resolution e.g. the examination of a cell nucleus, the Electron microscopic techniques such as the, Transmission Electron Microscope (TEM) where instead of using light to illuminate the specimen, a high energy electric beam is used. The scanning electron microscope (SEM), reflection electron microscope (REM), scanning transmission electron microscope (STEM), and the cryo-electron microscopy (Cryo-EM) are other forms electron microscopes techniques each with the unique method for how the structure and composition information is gained from the object [155–159]. Cryo-EM especially has produced very useful insights by enabling the determination of atomic resolution level macromolecular structures [160–162]. In protein structure research, Cryo-EM has been used to capture protein structure in its native state. Some methods have been developed to complement the microscopy techniques. Ou et al. [163] combined electron microscopy with a labeling method to reveal the 3-D organization across multiple scales in the cell nucleus They developed a method called ChromEMT that reveals the 3-D packing of the DNA in cells, and through their method reveled information about the DNA folding as it relates to the genome compaction in the cell nucleus.

For many years, the FISH and the microscopy-based techniques have given scientists insight about the spatial organization and architectural arrangement in the nucleus while providing explanation for nuclear positioning in the cell nucleus. Some of these findings include: the discovery of chromosome territories [2], the organization of gene clusters and their influence on transcription in the nucleus [51, 52], the segmentation of chromatin in the cell nucleus, for example, the active euchromatin and inactive heterochromatin occupy separate environments in the nucleus [164], and the existence of unique compartments that influences functional interaction [165–168].

These methods provide valuable information regarding the genomes organization that can be used as base information when constructing models with Hi-C data. For example, it is common practice to use the results of FISH experiments as validation for chromatin conformation models generated by Hi-C experiments. This can be done by verifying that the spatial distances observed between multiple FISH probes are consistent with the predicted distance between the corresponding genome bins found in the Hi-C conformation model.

Despite the advancements in the FISH and the microscopy approaches, they are limited to studying a region of genome, and do not provide a universal and comprehensive view of the 3-D genome architecture [169] of the whole genome. The need to study the genomic organization at a genome wide scale led to the development of the chromosome conformation capturing techniques. However, it is worth noting that the chromosome conformation capturing techniques and the imaging techniques of probing genome/chromosome structures are complementary and the latter can experimentally validate the former.

## Summary and Future Insights

Our review of the methods for reconstructing the 3-D structure of the chromosome and genome has revealed that these methods can be largely categorized into three groups (distance-based methods, contact-based methods and probability-based methods) according to how IF is modeled. For each category, we have discussed their potential strength and weakness in reconstructing 3-D chromosome and genome structures. Although we have primarily grouped methods based on IF modeling, there are other ways they could be categorized. For instance, their classification could be based on the type of structure they generate [72, 78]. Methods that generate a single representative structure for a Hi-C dataset are consensus-based methods [66, 67]. Those that generate a variety of structures to represent the heterogeneity of Hi-C data are ensemble-based methods [45, 48]. Finally, population-based methods [68, 86, 89] generate a population of structures that, as a whole, is statistically consistent with the Hi-C data.

Despite the improvement in 3-D structure modeling approaches, the lack of a real structure with which to contrast these models remains a challenge. In particular, it is currently difficult to confirm the true modeling capability of 3-D genome methods. Although the introduction of 3-D-FISH data and Hi-C data for joint modeling has received some attention recently [94], there is no sufficient 3-D-FISH data to guide most modeling on Hi-C data and to thoroughly validate the quality of computational models. The development of more advanced genome/chromosome imaging techniques will further improve the validation of 3-D genome models. In addition, other high-throughput sequencing data such as functional genomics and epigenomics data can be used to validate the biological validity of 3-D genome/chromosome models by exploring their correlation with 3-D genomes.

Another challenge is to reconstruct high-resolution 3-D models of large genomes from Hi-C data,

which are needed for studying detailed interactions between genes and regulatory elements, due to enormous time complexity and data sparsity associated with high-resolution modeling. Only a few methods [98] was designed to build high-resolution (e.g. 5 KB) models.

Finally, it is important to make 3-D genome modeling methods easy for biomedical scientists to use in their research. To this end, a few tools have been designed to visualize 3-D genome models [88, 89, 170–174]. Recently, GenomeFlow [40] provides a comprehensive graphical environment for users to process Hi-C data, generate chromosomal contact maps, build 3-D models, and apply 3-D models to integrate various omics data. More efforts of making 3-D genome modeling accessible to general users are still needed.

## Abbreviations

3C: Chromosome conformation capture
3-D: Three-dimensional
CNS: Crystallography & NMR System
dPCC: Distance Pearson correlation coefficient
dRMSE: Distance root mean square error
dSCC: Distance Spearman’s correlation coefficient
FISH: Fluorescence in situ hybridization
Hi-C: The name of a method an extension of 3C method that is capable of identifying read pair interactions on an “all-versus-all” basis—that is, it can profile interactions for all read pairs in an entire genome
IF: Interaction frequency
IMP: Integrative Modeling Platform
LAD: Lamina-associated domain
MDS: Multidimensional scaling
NMDS: Non-metric multidimensional scaling
TAD: Topologically associated domains
TCC: Tethered conformation capture
